# Clinical characteristics, pregnancy outcomes and ovarian function of pregnancy-associated breast cancer patients: a retrospective age-matched study

**DOI:** 10.1186/s12885-022-09260-6

**Published:** 2022-02-07

**Authors:** Qiuyue Liao, Dongmei Deng, Qin Xie, Xiaoqin Gong, Xiaolin Meng, Yun Xia, Jihui Ai, Kezhen Li

**Affiliations:** 1 Department of Gynecology and Obstetrics, Tongji Hospital, Tongji Medical College, Huazhong University of Science and Technology, Wuhan, 430030 Hubei China; 2 Department of Thyroid and Breast Surgery, Tongji Hospital, Tongji Medical College, Huazhong University of Science and Technology, Wuhan, 430030 Hubei China

## Abstract

**Background:**

Pregnancy-associated breast cancer (PABC) is a rare disease with increasing incidence. The prognosis, pregnancy outcomes and subsequent ovarian function of PABC patients are attracting attention.

**Methods:**

Sixty-three PABC patients and 126 age-matched non-PABC patients were obtained in Tongji Hospital from January 2011 to September 2019. The clinical characteristics and ovarian function of PABC patients were compared with those of non-PABC patients. The pregnancy outcomes and neonatal outcomes of patients with breast cancer diagnosed during pregnancy (BCP) were described. Nonparametric tests, the χ2-test Kaplan–Meier, Cox regression and binomial logistic regression were used for analysis.

**Results:**

PABC patients were diagnosed with a more advanced tumour stage (II: 47.6% vs. 45.2%, III: 33.3% vs. 19.8%, IV 3.2% vs. 0%, *p* = 0.003), which caused worse progression-free survival (PFS) (log-rank *p* = 0.0138) and breast cancer-specific survival (CSS) (log-rank *p* = 0.0076) than non-PABC patients. Tumour stage (III/IV vs. 0/I/II) (HR 16.017, 95% CI 5.830 ~ 44.006, *p* < 0.001) and endocrine therapy (HR 0.254, 95% CI 0.099 ~ 0.653, *p* = 0.004) were predictors of PFS. Tumour stage (III/IV vs. 0/I/II) (HR 30.875, 95% CI 7.232 ~ 131.820, *p* < 0.001), endocrine therapy (HR 0.200, 95% CI 0.049 ~ 0.818, *p* = 0.025) and targeted therapy (HR 0.143, 95% CI 0.028 ~ 0.743, *p* = 0.021) were predictors for breast CSS. Among the 15 BCP patients, 11 patients voluntarily continued their pregnancy, and the newborns had no obvious birth defects, either in 5 patients who received chemotherapy or in 6 patients who did not receive chemotherapy during pregnancy. Among the patients who received chemotherapy and did not receive endocrine therapy, 24 PABC patients and 48 non-PABC patients experienced chemotherapy-induced amenorrhea. There was no significant difference in resumption of menstruation between the two groups at 6 months and 12 months after the end of chemotherapy. No potential factors affecting resumption of menstruation were found.

**Conclusion:**

Pregnancy at diagnosis or within 1 year after delivery was not a risk factor for a worse prognosis in PABC patients. Compared with non-PABC patients, patients with PABC presented more aggressive tumour characteristics, which could mostly explain the worse prognosis observed in PABC patients. Receiving the appropriate regimen of chemotherapy in the second and third trimesters did not affect the maternal outcomes or neonatal outcomes of BCP patients. The special physiological state during pregnancy and lactation did not interfere with the damage of chemotherapy to ovarian function.

**Supplementary Information:**

The online version contains supplementary material available at 10.1186/s12885-022-09260-6.

## Introduction

Pregnancy-associated breast cancer (PABC) is defined as breast cancer that is diagnosed during pregnancy and/or the postpartum period. The duration of the postpartum period, which ranges from 1 to 5 years, is controversial, but the most common is 1 year after delivery [1–4]. PABC is a rare disease with an incidence of 0.6% ~ 2.1% in terms of breast cancer, of 1 in 10,000 to 1 in 3000 in terms of pregnancy, and of the second most frequent types of pregnancy-related cancer [5, 6]. The incidence of PABC increases as the trend to postpone childbearing and the general increase in the incidence of breast cancer [7–9].

Many studies have indicated that PABC patients differ from non-PABC patients in clinicopathologic characteristics. For example, patients diagnosed with PABC are younger, suffer a more advanced stage, have larger tumour sizes and have more axillary lymph node metastasis than non-PABC patients [3, 4]. The advanced TNM stage at diagnosis of PABC patients might be caused by high levels of oestrogen and progesterone during pregnancy and high levels of prolactin during lactation or delayed diagnosis that was due to physiological changes of the breast during pregnancy or lactation and pathological changes of breast cancer [10, 11]. Studies about the most frequent molecular subtype in PABC did not reach a consistent conclusion, most studies about European and American PABC patients showed triple-negative breast cancer (TNBC) was the most frequent one, some studies about Asian PABC patients showed that Luminal B was the most frequent molecular subtype [12–14]. In addition, some studies showed that PABC was highly correlated with gene mutations, such as BRCA1/2 [15].

Considering that more advanced stage, larger tumour sizes, more axillary lymph node metastasis, and TNBC and BRCA1/2 mutations were associated with poor prognosis [16, 17], it is reasonable to infer that the prognosis of patients with PABC is worse than non-PABC patients. There have been many articles about the prognosis of PABC patients, but no consensus has been reached. Some studies reported that compared with non-PABC patients, the prognosis of PABC patients was worse, and their disease progression-free survival (PFS) and overall survival (OS) were worse than those of non-PABC patients of the same age [18]. Some studies showed that compared with non-PABC patients of the same age and similar diagnosis time, although PABC patients showed more aggressive tumour characteristics at the time of diagnosis, the prognosis of the two groups of patients was similar after adjusting for the relevant tumour characteristics [18]. However, Strasser-Weippl K et al. thought that after adjusting for possible prognostic factors, PABC patients of any age still had a worse prognosis than non-PABC patients of the same age [19].

Considering the positive impact of timely antitumor treatments on the prognosis of patients with breast cancer diagnosed during pregnancy (BCP) and the possible negative impact on fetal health, the management of BCP patients faces many challenges to balance those conflicts [3]. Some studies reported that compared with infants of BCP patients who did not receive chemotherapy during pregnancy, infants of BCP patients who received chemotherapy during pregnancy had a lower birth weight and more complications, specifically small for gestational age and neonatal intensive care unit (NICU) admission [20]. Some studies thought that under strict management, chemotherapy during pregnancy did not cause obvious fetal complications, which implied that fetal adverse events were associated with preterm birth but not chemotherapy that BCP patients received [21, 22]. The existence of controversy regarding the safety of chemotherapy during pregnancy in infants or the difference in treatments during pregnancy that cause different neonatal outcomes requires more clinical data for support and explanation.

It is widely known that chemotherapy is an important means of breast cancer treatment. A prominent side effect of chemotherapy is damage to ovarian function, leading to premature ovarian failure (POF) [23]. Most studies have shown that breast cancer patients who receive chemotherapy, especially cyclophosphamide and doxorubicin, have impaired ovarian function, manifesting as premature amenorrhea, increased levels of serum FSH, decreased levels of serum E2 and AMH and decreased antral follicle counts (AFCs) [24, 25]. In view of the current known main mechanisms of chemotherapy drugs on ovarian damage, the generally recognized main mechanism is the depletion of primordial follicles and the direct killing effect on growing follicles and primordial follicles [26, 27]. There is no relevant research reported on the damage to ovarian function caused by chemotherapy in PABC patients. It is well known that the ovaries are in a nonovulating state with no growing follicles during pregnancy and early lactation. Therefore, is the effect of ovarian damage in PABC patients less severe than that in non-PABC patients? Whether this special physiological state during pregnancy and lactation will have a protective effect on chemotherapy-induced ovarian damage urgently needs clinical evidence to be confirmed.

Therefore, the clinical characteristics of Chinese PABC patients and the impact of related treatments on pregnancy outcomes and ovarian function need to be further explored. Here, we report the clinical characteristics of 63 PABC patients and the pregnancy outcomes of 11 BCP patients and compare the resumption of menstruation of 24 PABC and 48 non-PABC patients who had chemotherapy-induced amenorrhea to verify the factors that impact the ovarian function of PABC patients.

## Materials and methods

### Patients

This retrospective study screened women aged 20 to 45 years old diagnosed with primary breast cancer between January 2011 and September 2019 in Tongji Hospital. In this study, the criteria were as follows: (1) newly treated cases in Tongji Hospital; (2) patients who had undergone surgery or pathological staging; (3) complete medical record information; and (4) follow-up duration after chemotherapy of more than 1 year.

Grouping criteria: ‘PABC’ was defined as breast cancer diagnosed during pregnancy or 1 year postpartum. ‘non-PABC’ is defined as breast cancer not diagnosed during that time. The non-PABC patients were matched with PABC patients with similar ages and times (less than 1 year) at diagnosis.

Clinical and demographic data were gathered from the patients’ medical records, including patient charts, operative and pathology reports, chemotherapy records, radiotherapy, endocrine therapy records and targeted therapy records, because BCP patients should include caesarean section operation records or delivery records. Data collection included the age at diagnosis, TNM stage, birth weight and Apgar score of newborns, date and type of surgery, radiotherapy, endocrine therapy, targeted therapy, and chemotherapy regimen and cycle numbers. Follow-up data on the date of recurrence and death, date of amenorrhea and resumption of menstruation were completed by telephone interview or multiple letters.

### Follow-up

Follow-up examinations were performed every 3 months for the first two years, then every 6 months for the following 3 years, and annually thereafter. The follow-up data were documented by the clinical research office. Definitions of disease recurrence included i) local if recurrence was ipsilateral; ii) regional if ipsilateral axillary recurrence; iii) distinct if metastasis to bone, live, brain, long or peritoneum, and for contralateral axillary recurrence. Progression-free survival was defined as the interval from the date of diagnosis to the date of recurrence or the last follow-up. Breast cancer-specific survival was defined as the time from diagnosis to death or the last date of follow-up. All deaths in our included cases were due to breast cancer recurrence or progression. Amenorrhea was defined as regular menstruation stopping more than 2 menstrual periods, which occurred during or after chemotherapy. Menstruation recovery was defined as two consecutive menstrual periods within 21–35 days.

### Statistical methods

Statistical Package for the Social Sciences (SPSS) 25.0 statistical software was used for statistical analysis. The distributions of clinicopathologic factors were evaluated using nonparametric tests or the χ2 test as appropriate. Progression-free survival and breast cancer-specific survival were estimated by the Kaplan–Meier model. A Cox proportional hazard regression model and binomial logistic regression model were used for multivariate analysis. Variables with *P* < 0.2 in univariate analysis were included in multivariate analysis. Differences were considered significant at *P* < 0.05.

### Ethics approval

This study was approved by the Ethics or Institutional Review Board of Tongji Hospital, Huazhong University of Science and Technology in Hubei Province (TJ-IRB20210306).

## Results

### Patient characteristics

In total, 63 women were diagnosed with PABC (15 patients were diagnosed during pregnancy, 48 patients were diagnosed postdelivery), and 126 non-PABC patients were included in our research after the age and year of diagnosis were matched with PABC patients at a ratio of 1:2.

The clinical and demographic data of the 63 PABC patients and 126 non-PABC patients are shown in Table 1. There was no significant difference in the percentage of patients who were diagnosed before or at 35 years old (71.4% VS 69.8%, *p* = 0.822), the median age at diagnosis (32 VS 33, *p* = 0.495), the median age at menarche (13 VS 13, *p* = 0.305), the family history of breast cancer or ovarian cancer (12.7% VS 7.9%, *p* = 0.293) or the childbearing history (88.9% VS 80.2%, *p* = 0.131) in PABC patients and non-PABC patients. Patients with PABC were more likely to be diagnosed at an advanced tumor stage (III: 33.3% VS 19.8%, *p* = 0.006) due to a larger tumor size (T2: 68.3% VS 45.2%; T3: 6.3% VS 2.4%, *p* < 0.001) and more axillary lymph node metastasis (N2: 22.2% VS 7.1%, *p* = 0.016) than non-PABC patients. The states of ER/PR/HER-2 of patients with PABC were similar to those of patients with non-PABC, as were the proportions of molecular subtypes.

**Table 1:** Clinicopathologic characteristics of patients with PABC and non-PABC

	PABC	non-PABC	*P*
Age at diagnosis			0.822
≤ 35	45(71.4%)	88(69.8%)	
> 35	18(28.6%)	38(30.2%)	
Median age	32	33	0.495
Median age at menarche	13	13	0.305
Family history of breast cancer			0.293
Yes	8(12.7%)	10(7.9%)	
No	55(87.3%)	116(92.1%)	
Childbearing history			0.131
Yes	56(88.9%)	101(80.2%)	
No	7(11.1%)	25(19.8%)	
Tumor size			** < 0.001**
Tis	0 (0%)	3(2.4%)	
T1	13(20.6%)	63(50.0%)	
T2	43(68.3%)	57(45.2%)	
T3	4(6.3%)	3(2.4%)	
T4	3(4.8%)	0(0%)	
Lymph node metastasis			**0.016**
N0	26(41.3%)	73(57.9%)	
N1	16(25.4%)	28(22.2%)	
N2	14(22.2%)	9(7.1%)	
N3	7(11.1%)	16(12.7%)	
Distant metastasis			0.209
M0	61(96.8%)	126(100.0%)	
M1	2(3.2%)	0(0.0%)	
TNM stage			**0.006**
0	0(0%)	3(2.5%)	
I	10(15.9%)	41(32.5%)	
II	30(47.6%)	57(45.2%)	
III	21(33.3%)	25(19.8%)	
IV	2(3.2%)	0(0%)	
ER			0.109
Positive	35(55.6%)	85(67.5%)	
Negative	28(44.4%)	41(32.5%)	
PR			0.061
Positive	30(47.6%)	78(61.9%)	
Negative	33(52.4%)	48(38.1%)	
Her-2			0.749
Positive	41(65.1%)	79(62.7%)	
Negative	22(34.9%)	47(37.3%)	
Ki-67			0.208
≥ 14%	53(84.1%)	96(76.2%)	
< 14%	10(15.9%)	30(23.8%)	
Molecular subtype			0.536
Luminal A	4(6.3%)	8(6.3%)	
Luminal B(Her-2 +)	23(36.5%)	58(46.0%)	
Luminal B (high ki-67)	9(14.3%)	22(17.5%)	
Her-2 overexpression	16(25.4%)	22(17.5%)	
TNBC	11(17.5%)	16(12.7%)	

In terms of treatments that patients received, as shown in Table 2, compared with patients with non-PABC, a larger percentage of PABC patients received neoadjuvant chemotherapy and adjuvant chemotherapy sequentially (39.7% vs. 10.3%, *p* < 0.001), and fewer PABC patients received endocrine therapy. There was no significant difference in the percentage of patients who received conservative breast surgery (14.3% vs. 19.0%, *p* = 0.416), radiotherapy (58.7% vs. 50.8%, *p* = 0.302) or targeted therapy (20.6% vs. 26.2%, *p* = 0.401) between PABC patients and non-PABC patients.

**Table 2:** Treatments of patients with PABC and non-PABC

	PABC	Non-PABC	*p*
Breast surgery			0.416
Conservative	9 (14.3%)	24 (19.0%)	
Mastectomy	54 (85.7%)	102 (81.0%)	
Chemotherapy			** < 0.001**
No	3 (4.8%)	6 (4.8%)	
NACT + ACT	25 (39.7%)	13 (10.3%)	
ACT	35 (55.5%)	107 (84.9%)	
Radiotherapy			0.302
Yes	37 (58.7%)	64 (50.8%)	
No	26 (41.3%)	62 (49.2%)	
Endocrine therapy			**0.049**
Yes	29 (46.0%)	77 (61.1%)	
No	34 (54.0%)	49 (38.9%)	
Targeted therapy			0.401
Yes	13 (20.6%)	33 (26.2%)	
No	50 (79.4%)	93 (73.8%)	

### Comparison of recurrence and survival between PABC and non-PABC patients

**Fig. 1:**
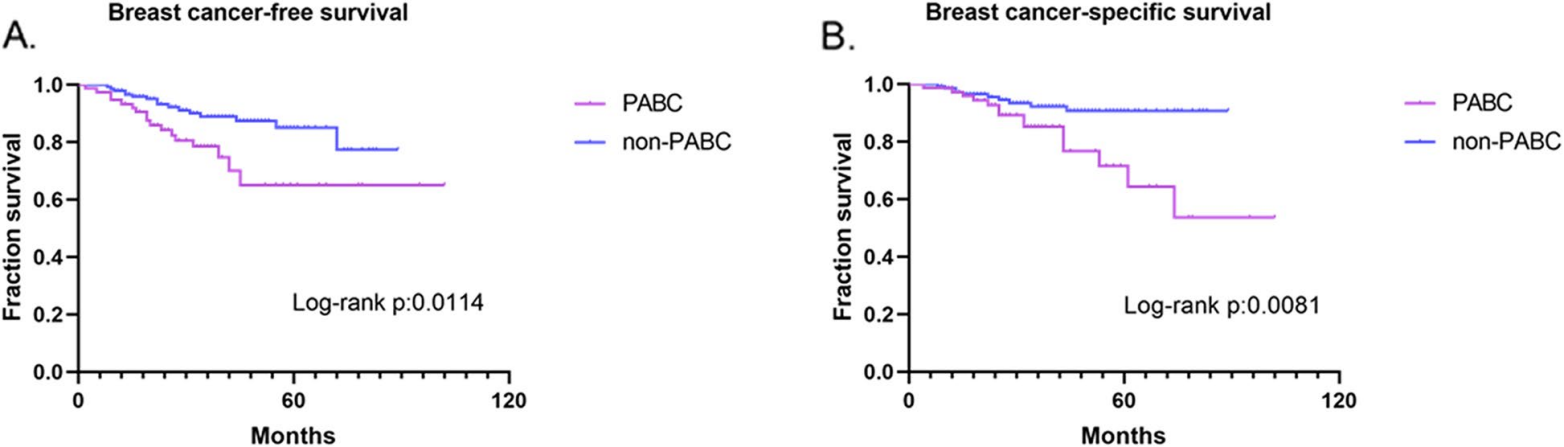
Kaplan–Meier analysis of survival in women with breast cancer. **A**, **B**, Progression-free survival and breast cancer-specific survival in women with PABC (*n* = 63) and non-PABC (*n* = 126)

The median follow-up was 33 months for PABC patients and 38 months for non-PABC patients. The median breast cancer-free survival was 32 months for the PABC group and 37.5 months for the non-PABC group. The recurrence rates were 25.4% (*n* = 16) and 12.7% (*n* = 16) in the PABC and non-PABC groups, respectively. The death rates were 20.6% (*n* = 13) and 7.9% (*n* = 10) in the PABC and non-PABC groups, respectively. As shown in Fig. 1, the Kaplan–Meier results of progression-free survival showed that patients with PABC had a worse prognosis than patients with non-PABC (log-rank *p* = 0.0138), as did the results of breast cancer-specific survival (log-rank *p* = 0.0076).

To explore whether pregnancy within 1 year of breast cancer diagnosis is a high-risk factor affecting the prognosis of patients, we performed univariate and multivariate Cox regression to adjust the confounding factors influencing the prognosis of patients with breast cancer (Tables 3 and  4).

**Table 3:** Multivariate Cox regression of factors influencing breast cancer-free survival in patients with PABC and non-PABC

Variable	level	Event	Total	Univariate mode			Multivariate model		
				HR	95%CI	*P*	HR	95%CI	*P*
PABC	Yes	16	63	2.340	1.164 ~ 4.701	**0.017**	0.971	0.437 ~ 2.158	0.943
	No	16	126	1					
Age at diagnosis	≤ 35	23	133	1.125	0.520 ~ 2.437	0.765			
	> 35	9	56	1					
Family history	Yes	6	18	2.430	0.997 ~ 5.925	**0.051**	2.204	0.831 ~ 5.844	0.112
	No	26	171	1					
TNM stage	III/IV	25	48	16.943	7.262 ~ 39.528	** < 0.001**	16.017	5.830 ~ ~ 44.006	** < 0.001**
	0/I/II	7	141	1					
ER	+	19	120	0.723	0.357 ~ 1.464	0.367			
	-	13	69	1					
PR	+	14	108	0.506	0.251 ~ 1.020	**0.057**	0.639	0.275 ~ 1.489	0.300
	-	18	81	1					
HR	+	19	124	0.652	0.322 ~ 1.321	0.235			
	-	13	65	1					
Her-2	+	25	120	2.261	0.976 ~ 5.234	**0.057**	1.568	0.652 ~ 3.774	0.315
	_	7	78	1					
Ki-67	≥ 14%	27	149	1.905	0.730 ~ 4.972	**0.188**	0.880	0.305 ~ 2.537	0.813
	< 14%	5	40	1					
Breast surgery	BMS	27	156	1.347	0.515 ~ 3.522	0.543			
	CMS	5	33						
Chemotherapy	No	1	9	1					
	ACT	17	142	0.844	0.112 ~ 6.363	0.870			
	NACT + ACT	14	38	4.177	0.545 ~ 32.002	0.209			
Radiotherapy	Yes	27	101	5.332	2.051 ~ 13.862	**0.001**	2.186	0.766 ~ 6.240	0.144
	No	5	88	1					
Endocrine therapy	Yes	10	106	0.278	0.131 ~ 0.588	**0.001**	0.254	0.099 ~ 0.653	**0.004**
	No	22	83	1					
Targeted therapy	Yes	5	46	0.643	0.247 ~ 1.673	0.366			
	No	27	143	1

**Table 4:** Multivariate Cox regression of factors influencing breast cancer-specific survive in patients with PABC and non-PABC

Variable	Level	Events	Total	Univariate model			Multivariate model		
				HR	95%CI	*p*	HR	95%CI	p
PABC	Yes	13	63	2.925	1.277 ~ 6.701	**0.011**	1.230	0.460 ~ 3.290	0.681
	No	10	126	1					
Age at diagnosis	≤ 35	16	133	1.009	0.414 ~ 2.456	0.985			
	> 35	7	56	1					
Family history	Yes	4	18	2.222	0.747 ~ 6.615	**0.151**	2.113	0.555 ~ 8.049	0.273
	No	19	171	1					
TNM stage	III/IV	18	48	15.741	5.810 ~ 42.646	** < 0.001**	30.875	7.232 ~ 131.820	** < 0.001**
	0/I/II	5	141	1					
ER	+	12	120	0.543	0.240 ~ 1.233	**0.144**	1766.341	0.000 ~ 3.509*10^7^	0.931
	-	11	69	1					
PR	+	10	108	0.530	0.231 ~ 1.216	**0.134**	1.111	0.174 ~ 7.081	0.912
	-	13	81	1					
HR	+	12	124	0.494	0.218 ~ 1.211	**0.092**	0.000	0.000 ~ 3.365*10^6^	0.920
	-	11	65	1					
Her-2	+	17	120	1.842	0.725 ~ 4.683	**0.199**	2.844	0.845 ~ 9.565	0.091
	-	6	69	1					
Ki-67	≥ 14%	19	149	1.813	0.612 ~ 5.371	0.283			
	< 14%	4	40	1					
Breast surgery	BMS	19	156	1.317	0.440 ~ 3.938	0.622			
	CMS	4	33						
Chemotherapy	No	1	9	1					
	ACT	13	142	0.529	0.068 ~ 4.089	0.542			
	NACT + ACT	9	38	2.141	0.270 ~ 16.958	0.471			
Radiotherapy	Yes	18	101	3.289	1.219 ~ 8.874	**0.019**	1.105	0.356 ~ 3.434	0.865
	No	5	88	1					
Endocrine therapy	Yes	4	106	0.134	0.045 ~ 0.394	** < 0.001**	0.200	0.049 ~ 0.818	**0.025**
	No	19	83	1					
Targeted therapy	Yes	2	46	0.356	0.083 ~ 1.520	**0.163**	0.143	0.028 ~ 0.743	**0.021**
	No	21	143	1					

In univariate COX proportional hazards regression for progression-free survival, patients with PABC (HR 2.340, 95%CI 1.164 ~ 4.701, *p* = 0.017), family history of breast cancer (HR 2.430, 95%CI 0.997 ~ 5.925, *p* = 0.051), more advanced TNM stage(III/IV vs. 0/I/II) (HR 16.943, 95%CI 7.262 ~ 39.528, *p* < 0.001), positive PR (HR 0.506, 95%CI 0.251 ~ 1.020, *p* = 0.057), positive Her-2 (HR 2.261, 95%CI 0.976 ~ 5.234, *p* = 0.057), larger Ki-67 (HR 1.905, 95%CI 0.730 ~ 4.972, *p* = 0.188), radiotherapy (HR 5.332, 95%CI 2.051 ~ 13.862, *p* = 0.001) and endocrine therapy (HR 0.278, *p* = 0.188), radiotherapy (HR 5.332.001) were significant factors that influenced progression-free survival, *p* = 0.001). Then, those factors were included in multivariate Cox analysis, and the results showed that a more advanced TNM stage (III/IV vs. 0/I/II) (HR 16.017, 95% CI 5.830 ~ 44.006, *p* < 0.001) was a predictor for poor breast cancer-free survival and patients receiving endocrine therapy (HR 0.254, 95% CI 0.099 ~ 0.653, *p* = 0.004) yielded better breast cancer-free survival outcomes (Table 3).

In univariate COX proportional hazards regression for breast cancer-specific survival, patients with PABC (HR2.925, 95%CI 1.277 ~ 6.701, *p* = 0.011), with family history of breast cancer or ovarian cancer (HR 2.222, 95%CI 0.747 ~ 6.615, *p* = 0.151), more advanced cancer stage (III/IV vs. 0/I/II) (HR15.741, 95%CI 5.810 ~ 42.646, *p* < 0.001), positive ER (HR 0.530, 95%CI 0.240 ~ 1.233, *p* = 0.144), positive PR (HR 0.530, 95%CI 0.231 ~ 1.216, *p* = 0.134), positive HR (HR 0.494, 95%CI 0.218 ~ 1.211, *p* = 0.092), positive Her-2 (HR 1.842, 95%CI 0.725 ~ 4.683, *p* = 0.199), radiotherapy (HR3.289, 95%CI 1.219 ~ 8.874, *p* = 0.019), endocrine therapy (HR 0.134, 95%CI 0.045 ~ 0.394, *p* < 0.001) and targeted therapy (HR 0.356, 95%CI 0.083 ~ 1.520, *p* = 0.163) were possible risk factors that influencing the outcomes of breast cancer-specific survival, as shown in Table 4. Then, those factors were included in multivariate Cox analysis, and the results showed that advanced TNM stage (III/IV compared with 0/I/II) (HR 30.875, 95% CI 7.232 ~ 131.820, *p* < 0.001) was a risk factor of breast cancer-specific survival, while endocrine therapy (HR 0.200, 95% CI 0.049 ~ 0.818, *p* = 0.025) and targeted therapy (HR 0.143, 95% CI 0.028 ~ 0.743, *p* = 0.021) were predictors for better breast cancer-specific survival outcomes (Table 4).

Pregnancy within 1 year of breast cancer diagnosis was not a risk factor that caused the difference in prognosis of patients with PABC and non-PABC. It is possible that the reason for the worse prognosis of PABC patients was that the tumour TNM stage of PABC patients was more advanced, so the prognosis was worse.

### Treatments during pregnancy and the related perinatal outcomes of BCP patients

**Fig. 2:**
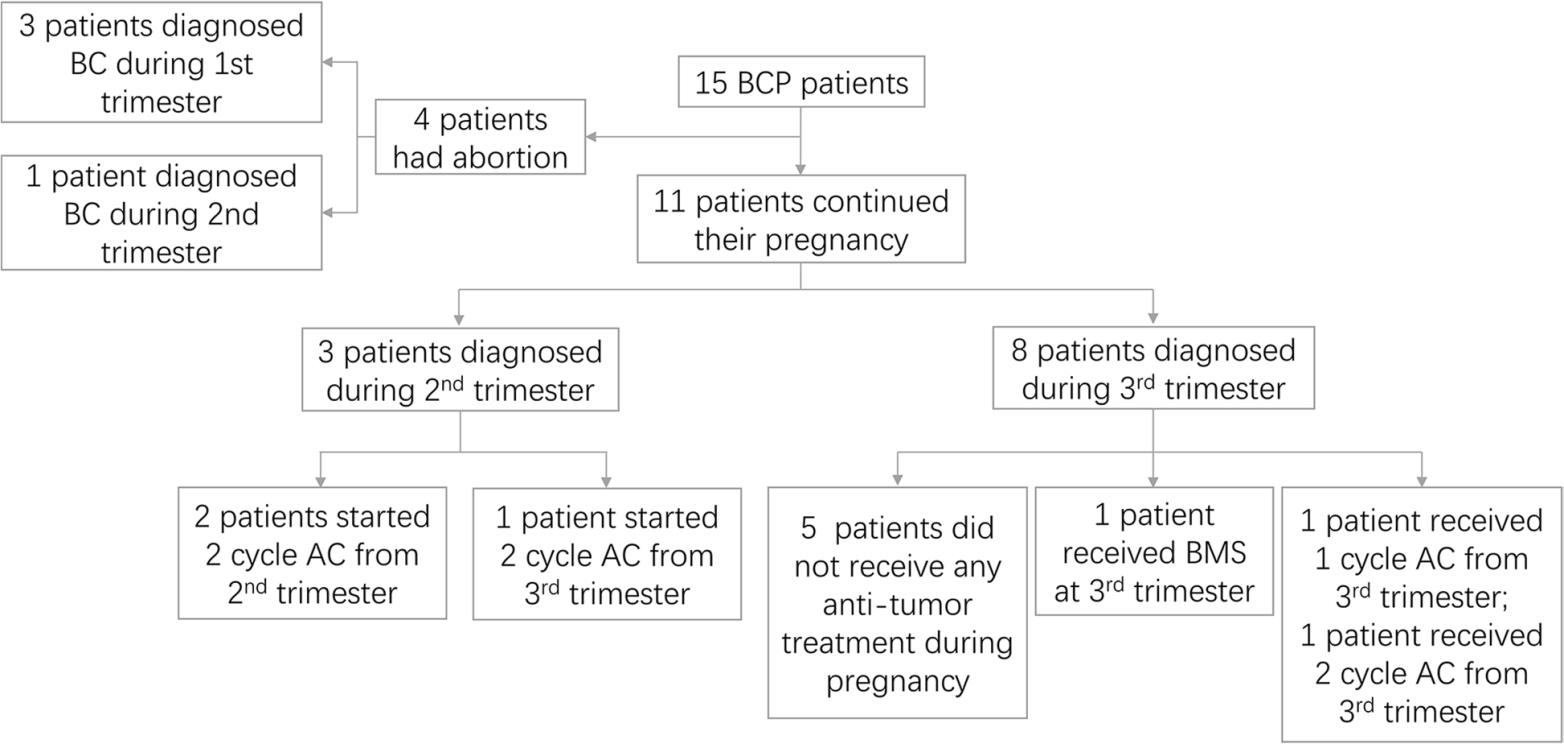
The Flowchart of included BCP patients

As shown in Fig. 2, only 15 BCP patients were obtained. Among the 15 BCP patients in this study, 4 patients (3 in the first trimester and 1 in the second trimester) were voluntarily aborted for starting antitumour treatments as early as possible, and 11 patients continued their pregnancy.

Among the 11 BCP patients, 4 (36.5%) received 2 cycles of AC (DOX + CTX) during pregnancy, 1 (9.1%) received 1 cycle of AC (DOX + CTX) during pregnancy, 1 (9.1%) underwent radical mastectomy in the third trimester, and the remaining 5 (45.5%) patients did not receive any antitumour therapy during pregnancy. Among the 5 BCP patients who received chemotherapy during pregnancy, 2 (20.0%) patients started NACT in the second trimester, and 3 (60.0%) patients started NACT in the third trimester.

As shown in Table 5, all 11 BCP patients delivered singleton live births successfully. Three patients had pregnancy complications during pregnancy, namely, hypertension during pregnancy, premature rupture of membranes, and central placenta previa. The Apgar scores at 1 min and 5 min of newborns of patients who received chemotherapy during pregnancy were similar to those of newborns of BCP patients who did not receive chemotherapy. Compared with the "Contrast Table of Newborns' Gestational Age and Weight", the weights of newborns in this study were all within the normal range, and there were no cases of infants smaller than gestational age.

**Table 5:** Perinatal outcomes of BCP patients who gave birth

ID	Maternal		Fetal				
	Treatments during pregnancy	Complications	gestational age(w)	Mode of delivery	Sex Of baby	Birth weight (kg)	Apgar score
01	BMS	None	37	Cesarean section	girl	2.5	8–8
02	1AC	None	34	Cesarean section	boy	2.3	8–8
03	2AC	None	35	Cesarean section	girl	2.4	8–9
04	2AC	PIH	34	Cesarean section	boy	2.2	8–9
05	2AC	None	37	Cesarean section	girl	2.6	8–7
06	2AC	None	34	Cesarean section	girl	2.3	8–9
07	None	PROM	31	Cesarean section	boy	2.1	7–7
08	None	PP	31	Cesarean section	girl	2.0	7–8
09	None	None	32	Cesarean section	boy	2.0	6–7
10	None	None	38	Normal delivery	girl	3.1	8–8
11	None	None	34	Cesarean section	girl	2.3	8–8

### Comparison of ovarian function between PABC and non-PABC patients who received chemotherapy

As a retrospective study, the results of hormones that reflected ovarian function could not be obtained. The resumption of menstruation is a clinical evaluation indicator of recovery of ovulation, and it is also an indirect reflection of the endocrine function of the ovaries after chemotherapy-induced amenorrhea. Therefore, we used the resumption of menstruation to represent the recovery of ovarian function. Considering the impact of endocrine therapy and history of menstrual disorders or surgery on menstruation, we excluded patients who received endocrine therapy, oophorectomy, or had a history of polycystic ovary syndrome. As shown in the supplementary Fig. 1, 24 PABC patients and 48 non-PABC patients who had chemotherapy-induced amenorrhea were included in this study.

**Fig. 3:**
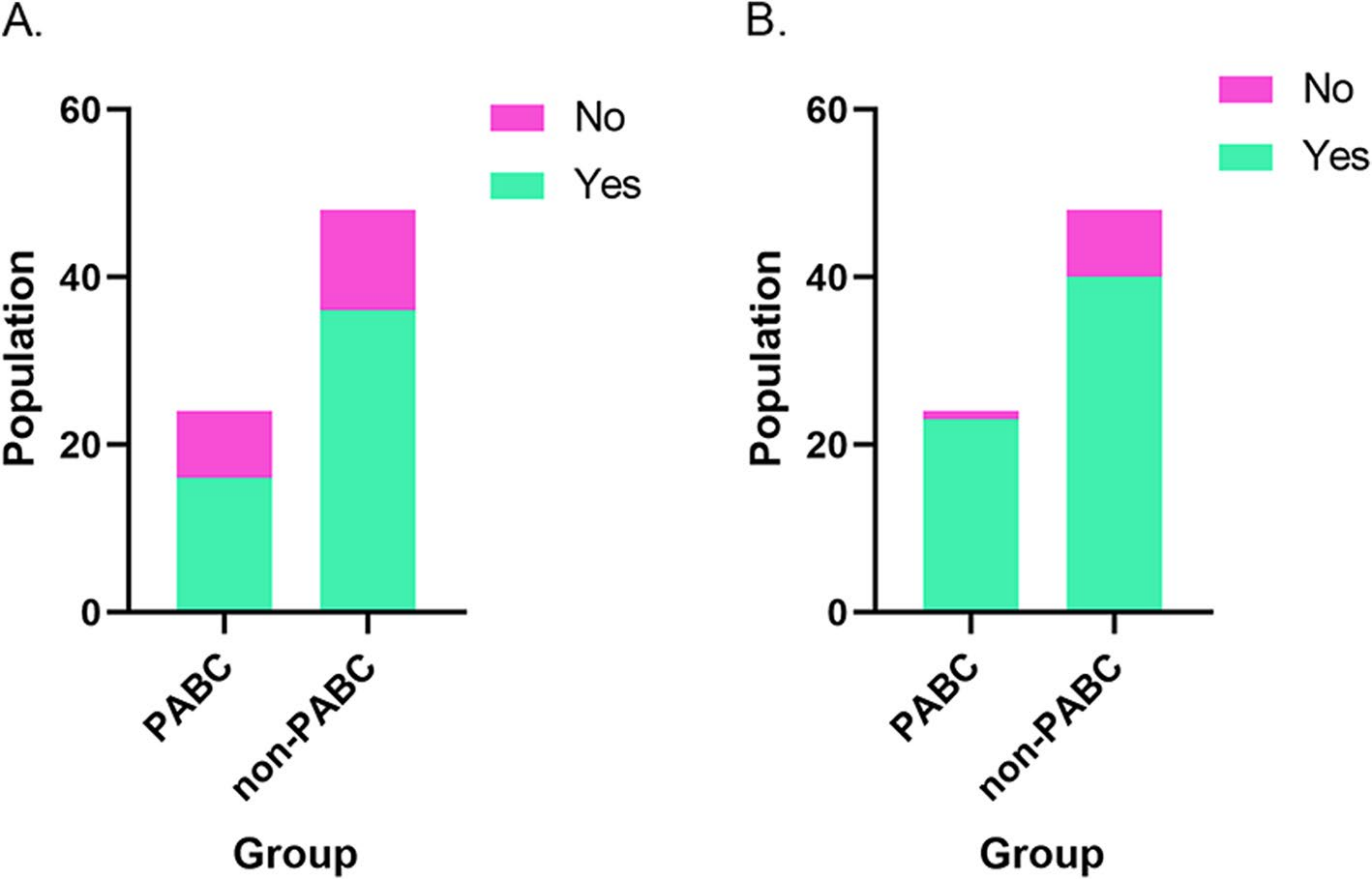
Bar graphs of resumption of menstruation at different times after chemotherapy of patients with PABC and non-PABC. **A**, At 6 months after ending chemotherapy, the resumption of menstruation of the PABC patients (*n* = 24) was similar to that of the non-PABC patients (*n* = 48). **B**, At 12 months after ending chemotherapy, the resumption of menstruation of the PABC patients (*n* = 24) was similar to that of the non-PABC patients (*n* = 48)

As shown in Fig. 3, the PABC patients’ and non-PABC patients’ rates of resumption of menstruation at 6 months after the end of chemotherapy were 66.7% (*n* = 16) and 75.0% (*n* = 36), respectively. The PABC patients’ and non-PABC patients’ rates of resumption of menstruation at 12 months after the end of chemotherapy were 95.8% (*n* = 23) and 83.3% (*n* = 40), respectively. There was no significant difference in the menstrual recovery rate between the two groups at 6 months and 12 months after ending chemotherapy (*p* > 0.05).

To explore the influencing factors of resumption of menstruation, we performed a binomial logistic regression model to adjust the confounding factors influencing menstruation of patients with breast cancer.

As shown in Table 6, patients with PABC, age at diagnosis, age at menarche, childbirth history, chemotherapy regimens, combined GnRH-a during chemotherapy, and targeted therapy, which might affect the resumption of menstruation, were included in binomial logistic regression. The above factors were not found to affect the resumption of menstruation at 6 months and 12 months after the end of chemotherapy (*p* > 0.05).

**Table 6:** Binomial logistic regression analysis of factors that may affect the resumption of menstruation after chemotherapy

	6 months after ending chemotherapy			12 months after ending chemotherapy		
	No resumption	non-resumption	p	No resumption	No. non-resumption	p
PABC			0.457			0.131
Yes	16	8		23	1	
No	36	12		40	8	
Age at diagnosis			0.744			0.691
≤ 35	37	15		46	6	
> 35	15	5		17	3	
Age at menarche			0.256			0.513
< 13	16	9		21	4	
≥ 13	36	11		42	5	
Childbearing history			0.638			0.633
Yes	44	16		53	7	
No	8	4		10	2	
Chemotherapy			0.556			0.365
8 (AC + D/T)	43	16		51	8	
6AC	4	3		7	0	
Others	5	1		5	1	
GnRH-a			0.352			0.131
Yes	33	15		23	1	
No	19	5		40	8	
Targeted therapy			0.630			0.203
Yes	16	5		20	1	
No	36	15		43	8	

## Discussion

Our study reported the clinical characteristics, current treatment statuses, prognostic factors and influence of treatment on pregnancy and ovarian function in Chinese patients with PABC. Compared with non-PABC patients, patients with PABC presented more aggressive tumour characteristics, including more advanced tumour stage and more axillary lymph node metastasis, which could mostly explain the worse prognosis observed in PABC patients. Receiving the appropriate regimen of chemotherapy in the second and third trimesters did not affect the maternal outcomes or neonatal outcomes of BCP patients. The special physiological state during pregnancy and lactation did not reduce the damage of chemotherapy to ovarian function.

Several studies reported that compared with non-PABC patients, PABC patients showed lower ER/PR expression and higher Her-2 overexpression [3, 28]. In contrast, in Chinese patients, when compared with non-PABC patients, PABC patients in our study had no statistically significant differences in the expression of ER/PR and Her-2. This suggests that there may be differences in disease characteristics between different races, so it is necessary to conduct a large-scale study of Chinese PABC patients.

Multiple studies have attempted to answer the question of whether pregnancy within 1 year before diagnosis is a negative factor for the prognosis of young patients with breast cancer [19, 29]. Due to different sample sizes and data sources, no consistent conclusions can be drawn. Inconsistently, another study that included 797 patients with PABC and 4177 age-matched patients with non-PABC, one of the maximum sample size studies to date, showed a 14% increased risk of death in patients with PABC even though the stage, race, and hormone receptor status were controlled [30]. Shao et al. systematically generalized 76 studies of the prognosis of patients with PABC, which showed that PABC was associated with poor prognosis for OS, disease-free survival (DFS) and CSS, and the pooled HRs with 95% CIs were 1.45 (1.30–1.63), 1.39 (1.25–1.54) and 1.40 (1.17–1.68), respectively [2]. Ploqun et al. reported that 111 patients from 27 centres with breast cancer diagnosed during pregnancy (PBC) had similar 5-year OS rates (83.1% vs. 85.5%, *p* = 0.31), with 253 non-PBC patients [31]. Anna’s research, with 778 PABC patients and 3598 non-PABC patients, reported that women with PABC had a similar survival as women with non-PABC after adjusting tumour characteristics in their age group [3], which was consistent with our results that the worse prognosis of PABC patients was mainly caused by more advanced tumour characteristics but not pregnancy within 1 year before diagnosis.

In our study, 5 BCP patients underwent NACT in the second and third trimesters, all of which were AC regimens. Only one patient underwent radical mastectomy in the third trimester. No neonates of patients who received NACT and mastectomy were found to have deformities, children below gestational age, or foetal distress. Our results are consistent with published articles. Studies have shown that during early pregnancy, especially during organogenesis, the use of chemotherapy drugs is related to foetal malformations [21, 32], but AC chemotherapy in the second and third trimesters of pregnancy has no significant negative effects on the foetus, and it is a widely recognized chemotherapy regimen during pregnancy [33]. According to NCCN guidelines [34], for BCP patients, mastectomy and axillary lymph node staging can be performed clinically in any trimester of pregnancy, chemotherapy can be administered during the second and third trimester, while radiotherapy and endocrine therapy should be administered after delivery [35, 36]. Therefore, receiving the recommended treatments at the appropriate gestational week during pregnancy is beneficial to the prognosis of BCP patients and does not significantly harm the health of the foetus.

To decrease the risk of recurrence, most patients with breast cancer receive adjuvant chemotherapy, which has been reported to result in temporary or permanent damage to ovarian function [37]. Resumption of ovarian function after long-term chemotherapy is important to subsequent quality of life in young patients with breast cancer [24]. Like most investigators, we used amenorrhea to represent the damage of ovarian function and the resumption of menstruation to indicate the recovery of ovarian function in our works [38, 39]. Our study reported that at 6 months and 12 months after the end of chemotherapy, the menstrual recovery rates of PABC patients and non-PABC patients were 66.7% and 75.0% (*p* > 0.05) and 95.8% and 83.3% (*p* > 0.05), respectively. The similar ovarian function of PABC and non-PABC patients after chemotherapy indicated that although PABC patients’ ovaries were in the anovulatory state with no growing follicles during pregnancy or early lactation, the protective effect was limited or useless and was not enough to weaken the ovarian damage caused by chemotherapy. We further analysed the potential factors affecting the resumption of menstruation in patients with breast cancer after chemotherapy. Although previous studies suggested that age, patients’ desire to have children, and utilization of GnRH agonists during chemotherapy could improve ovarian function [40, 41], no related factors were found in our study, and the small sample size may explain this.

There are some limitations to our study. First, this was a small retrospective study in a single centre. Our research provides the tumour characteristics and prognosis of Chinese patients with PABC, which is rarely mentioned in the existing literature. In the future, a large sample of Chinese PABC patients from multiple centres will be needed to verify the conclusions of our study. Second, it is partial to only use menstrual conditions to refer to ovarian function, and it should also include indicators such as AMH, AFC, FSH, E2 and so on. Since this study is a retrospective analysis, this part of the data is difficult to obtain. It was also due to this limitation that this study cannot evaluate the degree of damage or the recovery of ovarian function of PABC patients who had not recovered regular menstruation, and the latter was a more effective indicator for whether pregnancy or lactation could resist chemotherapy-induced ovarian damage. Third, the genotypes of the patients were not included in the survival analysis, mainly because some of the included cases were diagnosed at the time when genetic testing was not popular, thus lacking evidence of genotype. To obtain a credible result, a randomized controlled trial must be carried out in multiple centres.

## Conclusion

In conclusion, it is not pregnancy but the more aggressive tumour characteristics at diagnosis that cause the poorer prognosis of patients with PABC than that of non-PABC patients. Timely treatment during pregnancy is necessary because appropriate treatment at the right time during pregnancy will not negatively affect the foetus. The special ovulation state during pregnancy or after childbirth cannot effectively reduce the damage of chemotherapy to the ovaries.

## Supplementary Information

**Additional file 1.**

## Data Availability

The dataset supporting the conclusions of this article is available from the corresponding author upon reasonable request via tjkeke@126.com.

## Abbreviations

AFCs: Antral follicle counts
BCP: Breast cancer diagnosed during pregnancy
CSS: Cancer-specific survival
DFS: Disease-free survival
NACT: New adjuvant chemotherapy treatment
NICU: Neonatal intensive care unit
PABC: Pregnancy-associated breast cancer
PFS: Progression-free survival
POF: Premature ovarian failure
OS: Overall survival
SPSS: Statistical Package for the Social Sciences
TNBC: Triple-negative breast cancer
