# Supplementary figures and images for: Clinical characteristics, pregnancy outcomes and ovarian function of pregnancy-associated breast cancer patients: a retrospective age-matched study
Source: BMC Cancer. 2022 Feb 7;22:152. doi: 10.1186/s12885-022-09260-6 (PMC8822700; doi:10.1186/s12885-022-09260-6)

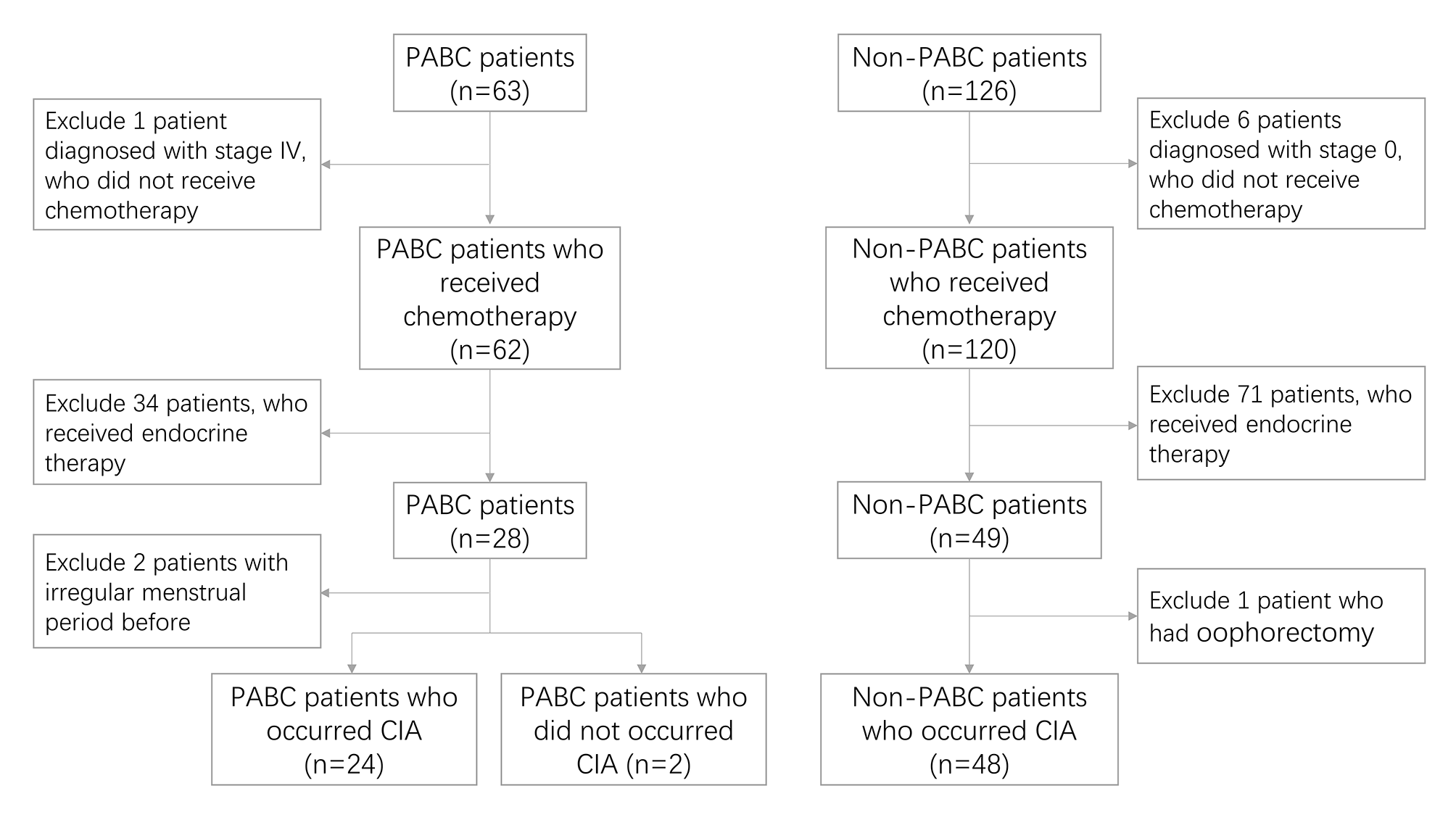

Supplement: Supplementary file 1 — Additional file 1. [file 12885_2022_9260_MOESM1_ESM.tif]
